# A unified framework for modelling sediment fate from source to sink and its interactions with reef systems over geological times

**DOI:** 10.1038/s41598-018-23519-8

**Published:** 2018-03-27

**Authors:** Tristan Salles, Xuesong Ding, Jody M. Webster, Ana Vila-Concejo, Gilles Brocard, Jodie Pall

**Affiliations:** 1 Geocoastal Research Group, School of Geosciences, University of Sydney, Sydney, NSW 2006 Australia; 2 Earthbyte, School of Geosciences, University of Sydney, Sydney, NSW 2006 Australia

## Abstract

Understanding the effects of climatic variability on sediment dynamics is hindered by limited ability of current models to simulate long-term evolution of sediment transfer from source to sink and associated morphological changes. We present a new approach based on a reduced-complexity model which computes over geological time: sediment transport from landmasses to coasts, reworking of marine sediments by longshore currents, and development of coral reef systems. Our framework links together the main sedimentary processes driving mixed siliciclastic-carbonate system dynamics. It offers a methodology for objective and quantitative sediment fate estimations over regional and millennial time-scales. A simulation of the Holocene evolution of the Great Barrier Reef shows: (1) how high sediment loads from catchments erosion prevented coral growth during the early transgression phase and favoured sediment gravity-flows in the deepest parts of the northern region basin floor (prior to 8 ka before present (BP)); (2) how the fine balance between climate, sea-level, and margin physiography enabled coral reefs to thrive under limited shelf sedimentation rates after ~6 ka BP; and, (3) how since 3 ka BP, with the decrease of accommodation space, reduced of vertical growth led to the lateral extension of reefs consistent with available observational data.

## Introduction

Most climate projections suggest that by 2100 sea level will have risen by 0.5–1.0 m^1^. Rainfall patterns will be significantly modified and occurrence and/or intensity of extreme events such as storms and tropical cyclones will increase^2^. It is not clear, however, how sediment dynamics and reef evolution might respond to these changes. Even though the actual rate of global warming far exceeds that of any previous episodes in the past 10,000 years, large changes in global climate have occurred periodically throughout Earth’s history^3^. Knowing how these past changes altered sediment transport from landmasses to coasts and how sediment accumulation influenced reef development may help us identify specific patterns and improve future predictions.

In the past, modelling efforts have focused mainly on the changes in regional and global wave climate, ocean biogeochemical cycles and sediment transport rates in response to projected climate-driven variations^4,5^. These complex models provide important insights on time scales of the order of 10 to 100 years, but cannot capture the cumulative effect of long-term, simulated processes (>1000 years). Additional problems lie in the computational resources and time required to run them. As a consequence, they are limited in applicability and cannot be ground-truthed over geological time scales (thousands to millions of years).

Bridging the gap between decadal to multi-millennial scales is numerically challenging and can only be solved using heuristic rather than classical methods. Our model is based on a hybrid of simplified governing equations for fluid motion and sediment transport which combines sediment dynamics induced by rivers and waves with a fuzzy logic method to simulate reef evolution. This reduced-complexity model enables us to quantitatively evaluate the role of climate, tectonics and sea level change on landscape dynamics, sediment delivery to the coasts, sediment accumulation on the shelf, slopes and offshore basins, wave-induced longshore transport and reef growth.

To our knowledge, previous regional-scale forward models have either been limited to one part of the sediment routing system^6–8^ (*e.g*. fluvial geomorphology, coastal erosion, carbonate platform development) or built upon simple laws commonly derived from diffusion-based equations, the application of which requires pluri-kilometric spatial resolution^9–11^. Therefore, our understanding of sediment transport (even at first-order) within and between the different Earth’s components is often limited. It also makes it difficult to link site-specific observations to numerical model outputs particularly in the case of reef systems. The model presented in this study, and the development efforts behind this unified framework, are intended to address these limitations. One of the most direct applications of our model is to better constrain sediment transport processes in mixed siliciclastic-carbonate systems. Studies of sediment cores from basins adjacent to the Great Barrier Reef (GBR) have challenged traditional models for offshore sedimentation on these types of mixed siliclastic-carbonate margins^12,13^. Our new model provides a more direct and flexible description of the inter-connectivities between land, shallow marine, reef and deep-water environments, in that it explicitly links these systems together at a spatial horizontal scale of a few hundred metres and a temporal scale of several thousand years. With typical runtime ranging from 3 to 5 hours on a standard computer, it also represents a promising basis for parameterisation of models versus field observations. First, we present the main physical components and describe the constitutive laws and underlying assumptions that drive sediment dynamics in our model. Then second, we model the 14,000 years of post-glacial evolution of sediment accumulation along the GBR and explore landscape erosion, sedimentation patterns and reef growth to assess the effectiveness of our approach.

## Methods

### Extrinsic forcings

At basin-scale, sediment transport and reef evolution are strongly controlled by large-scale forcings. Our model allows consideration of the following set of external forcing mechanisms: sea level variations, tectonic changes, rainfall regimes and boundary wave conditions. Spatial and temporal variations in precipitation can be applied as a set of maps representing an annual rainfall regime. The tectonic changes are provided as a series of temporal maps. Each map can have variable spatial cumulative displacements making it possible to simulate complex 3D tectonic evolution with both vertical (uplift and subsidence) and horizontal directions. The combination of these forcing mechanisms controls the evolution of the hydrodynamic conditions and the associated sediment transport regimes as well as marine carbonate production.

### Landscape evolution

We use the stream power law (SPL) to predict sediment transport in rivers^14^. This law relates erosion rate $\dot{\varepsilon }$ to drainage area *A* (which depends on net precipitation) and local river gradient *S* and takes the form:

1 $$\dot{\varepsilon }={\kappa }_{e}{A}^{m}{S}^{n}$$

where *κ*_*e*_ is an erodibility coefficient that depends on lithology and climate, while *m* and *n* are positive exponents^15^ that mostly depend on catchment hydrology and the nature of the dominant erosional mechanism^16^. Despite its simplicity, eq. 1 reproduces many of the characteristic features of natural systems where detachment-limited erosion regime dominates^17^.

In addition to overland flow, semi-continuous processes of soil displacement are accounted for using a linear diffusion law commonly referred to as soil creep^18,19^:

2 $$\frac{\partial z}{\partial t}={\kappa }_{d}{\nabla }^{2}z$$

in which *z* is the elevation and *κ*_*d*_ is the diffusion coefficient. This transport rate depends linearly on topographic gradient and encapsulates in a simple formulation the processes operating on superficial sedimentary layers.

### Wave transformation

We adopt the most basic known principles of wave motion, *i.e*. linear wave theory^20^, where wave celerity *c* is governed by:

3 $$c=\sqrt{\frac{g}{\kappa }\,\tanh \,\kappa d}$$

where, *g* is the gravitational acceleration, *κ* the radian wave number (equal to 2*π*/*L*, with *L* the wave length), and *d* is the water depth. From wave celerity and wave length, we calculate wave front propagation (including refraction) using the Huygens principle^21^. We then deduce the wave travel time and define wave directions from lines perpendicular to the wave front. Wave height (*H*) is then calculated along the propagating wave front. The algorithm takes into account wave energy dissipation in shallow environments as well as wave-breaking conditions. Given the long time scales of our simulations, we use a stationary representation of prevailing fair-weather wave conditions to evaluate marine sediment transport. At any given time interval, we define a percentage of activity for each deep-water wave conditions and the bathymetry is used to compute associated wave parameters.

Wave-induced sediment transport is related to the maximum bottom wave-orbital velocity *u*_*w*,*b*_. Assuming the linear shallow water approximation^22^, its expression is simplified as:

4 $${u}_{w,b}=(H\mathrm{/2)}\sqrt{g/d}$$

Under pure waves (*i.e*., no superimposed current), the wave-induced bed shear stress *τ*_*w*_ is typically defined as a quadratic bottom friction^23^:

5 $${\tau }_{w}=\frac{1}{2}\rho {f}_{w}{u}_{w,b}^{2}$$

where *ρ* is the water density and *f*_*w*_ is the wave friction factor. Considering that *f*_*w*_ is only dependent of bed roughness *k*_*b*_ relative to wave-orbital semi-excursion at the bed *A*_*b*_^24^, we define:

6 $${f}_{w}=1.39{({A}_{b}/{k}_{b})}^{-0.52}$$

where *A*_*b*_ = *u*_*w*,*b*_*T*/2*π* and *k*_*b*_ = 2*πd*_50_/12, with *d*_50_ median sediment grain size at the bed and *T* the wave period.

For each wave condition, the wave transformation model computes wave characteristics and the induced bottom shear stress. Our model uses these parameters to subsequently evaluate long-term sediment transport active over the simulated region.

### Long-term wave-driven sediment transport

We assume that flow circulation is mainly driven by waves^25^ and therefore we ignore other processes such as coastal upwelling and tide-, ocean- or local wind-driven currents. In nearshore environments, longshore currents run parallel to the shore and significantly contribute to sediment transport^26^. The longshore current velocity (*v&vec;*_*l*_) in the middle of the breaking zone is defined by^27^:

7 $${\overrightarrow{v}}_{l}={\kappa }_{l}{u}_{w,b}\,\cos (\theta )\sin (\theta )\overrightarrow{k}$$

with *θ* the angle of incidence of the incoming waves, *κ*_*l*_ a scaling parameter and *k&vec;* the unit vector parallel to the breaking depth contour. The calculation of *θ* is deduced from bathymetric contours and wave directions and requires an estimate of wave breaking depth.

In regions where wave-induced shear stress (eq. 5) is greater than the critical shear stress^28^ derived from the Shields parameter (*τ*_*c*_ = *θ*_*c*_*gd*_50_(*ρ*_*s*_ − *ρ*_*w*_)), bed sediments are entrained. The erosion thickness *h*_*e*_ is limited to the top sedimentary layer and for simplicity is assumed to follow a logarithmic form^29^:

8 $${h}_{e}={C}_{e}\,\mathrm{ln}({\tau }_{w}/{\tau }_{c})\,{\rm{where}}\,{\tau }_{w} > {\tau }_{c}$$

where *C*_*e*_ is an entrainment coefficient controlling the relationship between shear stress and erosion rate^29^. Once entrained, sediments are transported following the direction of longshore currents and are deposited in regions where *τ*_*w*_ < *τ*_*c*_^30^.

### Reef growth

We are still limited in our numerical representation of coral reef evolution^31^ owing to both the complex physical and biological interactions and the broad range of spatio-temporal scales involved. Additionally, most datasets on reef systems are often linguistic, context-dependent, and based on measurements with large uncertainties. Numerical methods such as fuzzy logic have proven to be a viable approach to simulate these types of systems^31^.

Based on fuzzy logic, reef evolution in our model is computed from a set of simple rules that control reef growth through space and time. The utility and effectiveness of the proposed approach is mostly based on the user’s understanding of the modelled reef system^11^. Here, reef growth depends on three types of basic control variables: depth (accommodation space), wave energy, and sedimentation rate. For each of these variables, one can define a range of fuzzy sets using membership functions^32^. The model also simulates multiple coral assemblages, representing specific groups of coral communities based on depth and/or environmental conditions. Production of any specific assemblage is then computed from a series of fuzzy rules which depend on the aforementioned control variables. Summation of multiple rules produces a fuzzy answer which is then defuzzified^33^ (using the centroid method) to return a value for coral assemblage growth in each cell of the simulated region. The same approach is also used to compute hemipelagic deposition based on an annual sedimentation rate that depends on both depth and wave energy.

## The GBR Holocene Settings

### Topography and bathymetry surfaces

**Figure 1:**
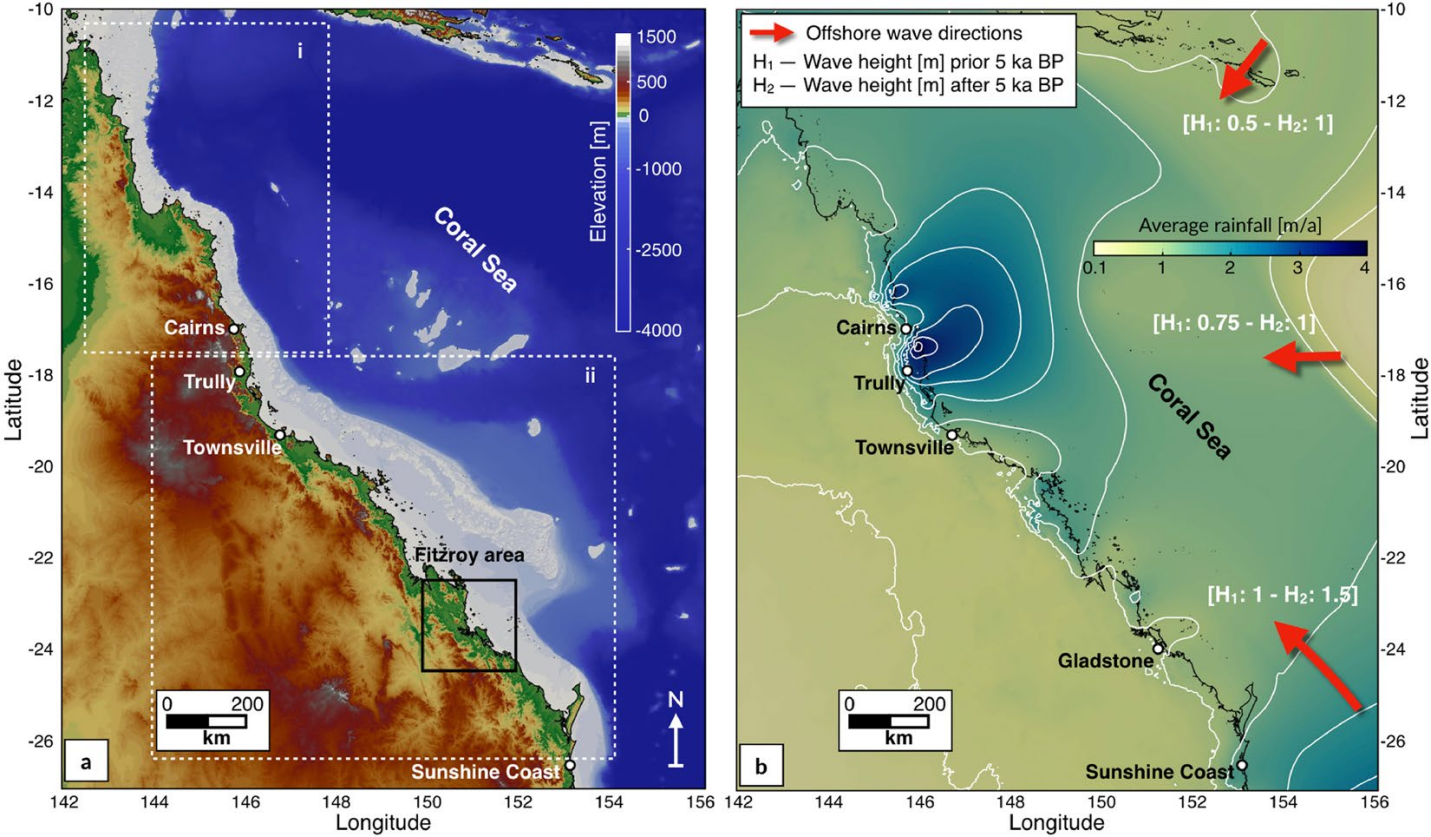
(**a**) Map shows the extend of the 2 regions (i-north, ii-south) of the GBR used in this study (source: Project 3DGBR-eAtlas.org.au). (**b**) Background map shows the average rainfall annual distribution based on 30-year records (1961–1990) encompassing several ENSO events (7 El Niño - 5 La Niña) (source: Bureau of Meteorology). White lines highlight precipitation 0.5 m/a contours. Red arrows define prevailing annual offshore wave directions scaled based on their annual activity. Wave heights (H) imposed for the considered 2 climatic scenarios from 14 to 5 ka and from 5 ka to present. Both maps were generated using Paraview (V 5.2.0).

The initial surface used in the model is based on a 100 m resolution grid^34^ that combines high-resolution bathymetry and a land digital elevation model. The dataset was resampled to a resolution of 500 m and divided in 2 regions (i and ii as shown in Fig. 1, containing ~1.5 and 2.9 million cells respectively). Care was taken to ensure that the entire area of catchments contributing sediments to the coast were included in these regions. To construct the paleo-surface we further modified the bathymetry to account for *(1)* sediment accumulation along the coast and the inner shelf and *(2)* coral reef development since the Holocene.

During the early Holocene, steady sea level rise resulted in the westwards migration of the shoreline preventing the accumulation of large terrigenous deposits along the shelf. Coincident with sea level reaching high-stand (~6 ka), the pattern of deposition began to stabilise. Consequently, most of the Holocene terrigenous sedimentation within the GBR can be assumed to be predominantly confined to the inner shelf and broadly extends to no farther than the 20–25 m isobath. Sedimentation rates along the Queensland coastline are highly variable^25^, with thickness of the inner shelf Holocene sediment wedge ranging from less than 5 m^35^ to 15 m^36,37^. To construct our initial paleo-surface to run the model, we assumed an average maximum 7.5 m deposition along the entire coast at around the 15 m isobath, tapering to 0 m at the coast and at the 25 m isobath, and removed this estimated sediment wedge from the initial surface. The paleo-surface is then further refined by removing the average thickness of the Holocene reefs. Details of reef cores reaching the Pleistocene surface in the GBR show variations in reef thicknesses from 5 to 25 m^38,39^. These regional variations have been inferred to depth, size and shape of the underlying antecedent surface^40^. To remove the Holocene coral reefs, we first obtained the modern reef positions and remove estimated Holocene coral reef thicknesses in these locations.

### Precipitation

Sediments delivered to the coast are primarily transported by river systems (*e.g*. the Fitzroy and the Burdekin)^41^. Estimates of modern annual sediment supply to the shelf from these catchments are about 13–28 Mt^42^. Rainfall varies substantially across the region and occurs mainly during Austral summer (from November to April). Mountains along the escarpment between Cairns and Trully receive the highest rainfall, owing to orographic effects (Fig. 1). Climatic reconstructions of Holocene precipitation variations^43,44^ generally show similar evolution attributed to the precessional control of the Walker circulation in the Pacific and the monsoon intensification. These trends have also been identified from analysis of series of high-resolution pollen records^45^ and chronostratigraphic dataset^46^. Despite discrepancies in chronologies, these observations suggest three distinct periods. From the beginning of the Holocene until ~6 ka, precipitation gradually increased. Between ~6 to ~4 ka, precipitation reached a maximum. Finally, from ~4 ka to present, precipitation decreased sharply, indicative of the onset of present-day ENSO dynamics^46^. We use the 30-year average rainfall map (Fig. 1) as a proxy for regional distribution of precipitation patterns. This assumption is valid as trade-winds have not changed significantly in direction^45^ and because orographic effects in the past would have certainly caused local rainfall anomalies similar to the ones observed at present day. To reflect the change in deglacial and Holocene rainfall amplitudes, we scaled down today’s rainfall intensity by half at 14 ka and increased it incrementally up to 6 ka. From 6 to 4 ka, the precipitation was kept constant as 1.5 times today’s rainfall. From 4 ka to present, we imposed a linear decrease towards present day rainfall values.

### Sea level and wave regime

The Australian region was relatively stable tectonically over the simulated period^47^, therefore, sea level changes represent the main factor controlling sediment accumulation across the GBR shelf. Holocene sea level variability around Australia has been the focus of many studies since the early compilations by Hopley^48^ and Chappell^49^ to the most recent works from Lewis^47^ and Leonard^50^. There is a general consensus in regards to the timing and magnitude of mid-Holocene sea level highstand^47^. However, the evidence is contradictory about the timing of the late Holocene sea level fall. Here, we used the sea level curve from Lambeck *et al*.^51^. From 14 to ~8.2 ka, the average rate of rise was about 12 m/ka and progressively decreased to ~2.5 ka after which ocean volumes remained nearly constant.

Offshore wave conditions for the GBR are well documented and usually derived from hind-cast models^52^, but data for the inner shelf are scarce and limited to a few local studies where *in-situ* wave measurements were obtained^53^. Seaward of the GBR, mean, peak wave period is generally between 8 and 9 s and along the 2,000 m contour, mean significant wave height vary between 1 and 2 m (Fig. 1). Coral Sea dominant wave direction is southeasterly and follows the trade-winds, which blow persistently in the region throughout winter. In summer, a reversal of wind direction induces variable east-northeast waves^52^ and occasionally, cyclone-generated waves^37^.

Holocene wave regimes are still poorly constrained for the GBR. However, observations of fossil reef cores from several locations^39,54^ indicate that both low and high energy corals communities have co-existed across the outer reefs since the mid-Holocene. Comparisons between sea level rise and reef growth also indicate that wave energies have been relatively stable over the last ~5 ka^36^. During early-mid Holocene time (prior to 5 ka), studies have shown that ENSO was significantly weakened and speculate that the later shift in stronger ENSO intensity was associated to an increase in wave energy^55^. Following these observations, wave propagation in our study was forced by two climatic scenarios (Fig. 1). Prior to 5 ka, offshore wave heights were distributed along the southeast [annual activity (AA): 60%, wave height (H): 1 m], east [AA: 20%, H: 0.75 m] and northeast [AA: 20%, H: 0.5 m] directions. After 5 ka, offshore wave directions followed the same pattern but wave heights increased to 1.5 m (southeast) and 1 m (east and northeast).

## Results

### Landscape erosion

**Figure 2:**
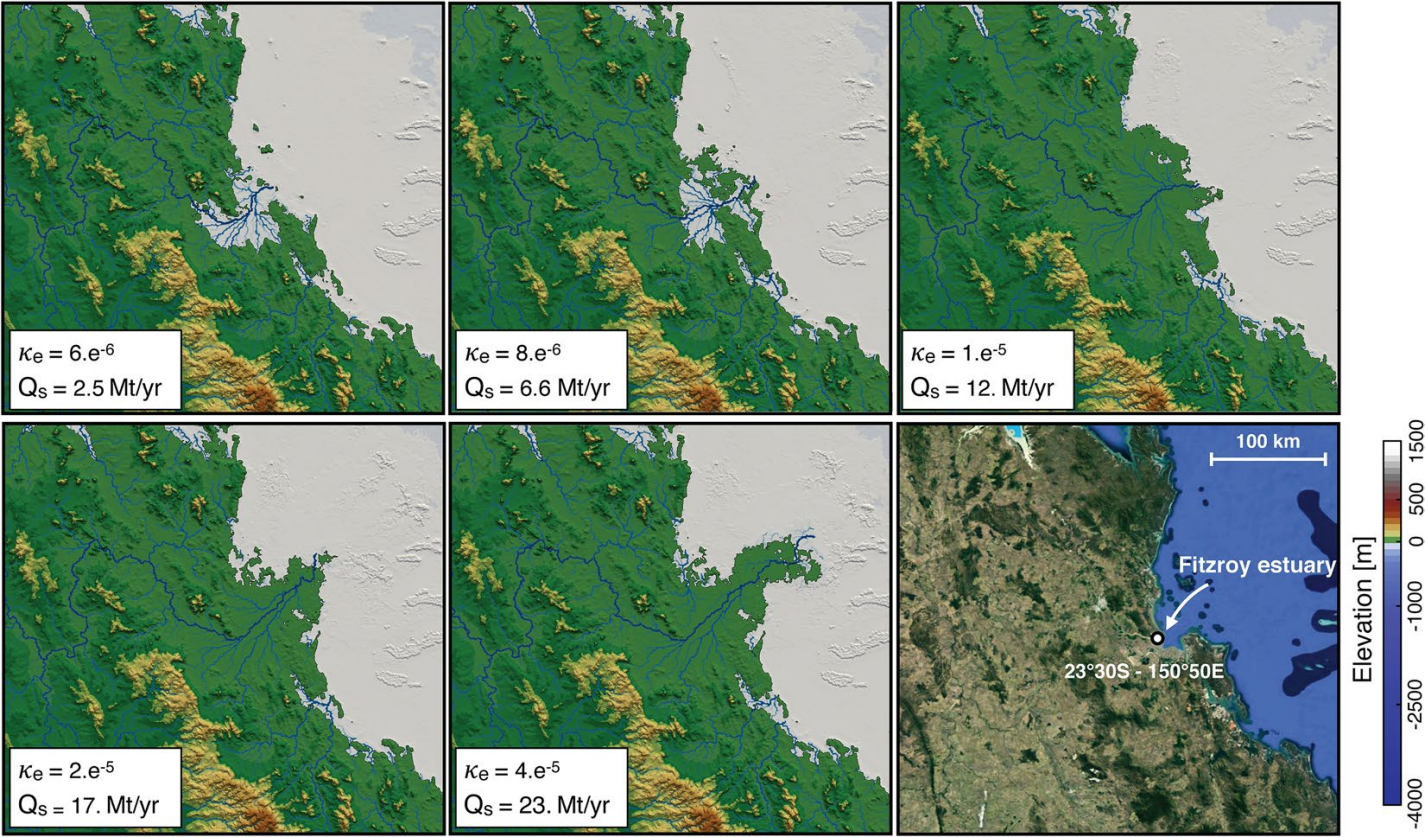
Sensitivity analysis of erodibility coefficient (*κ*_*e*_ in eq. 1) at final time step for 5 simulations showing its effects on sediment accumulation (Mt/yr) and Fitzroy delta progradation (Fitzroy area is shown in Fig. 1a) visualised with Paraview (V 5.2.0). Lower right panel presents today’s topography from Map Data @2018 GBRMPA, Google.

The simulations presented in this paper result from a series of sensitivity tests on the main physical parameters described in the methods. Figure 2 shows how the coefficient of erodibility has been calibrated. The SPL formulation (eq. 1) depends on *κ*_*e*_, *m* and *n* which cannot easily be measured from direct field observations. Here, we set the values of *m* and *n* to 0.5 and 1 respectively and we deduce best fit erodibility value from observational constraints. Although there are 35 river catchments draining into the GBR, two thirds of the terrigenous sediment (19.6 Mt/a) is contributed by the Burdekin and Fitzroy River catchments alone^56^. Using the Fitzroy catchment, we tested values of *κ*_*e*_ ranging from 6.e^−6^ to 4.e^−5^/yr and compared the results both in terms of extent of the sediment depositional area and fluxes delivered to the coast (*Q*_*s*_ in Fig. 2). Average sediment fluxes were computed using cumulative erosion over the last period of 500 years for the entire catchment assuming a mean sediment density of 2650 kg/m^3^. Reported field estimates of annual sediment discharge^57^ are highly dependent of precipitation and range between 2 and 10 Mt/yr. From the tested values, present day estuary morphology (bottom right panel in Fig. 2) and sediment fluxes, observations are better reproduced with a *κ*_*e*_ of 8.e^−6^/yr.

### Sediment accumulation and reef evolution

**Figure 3:**
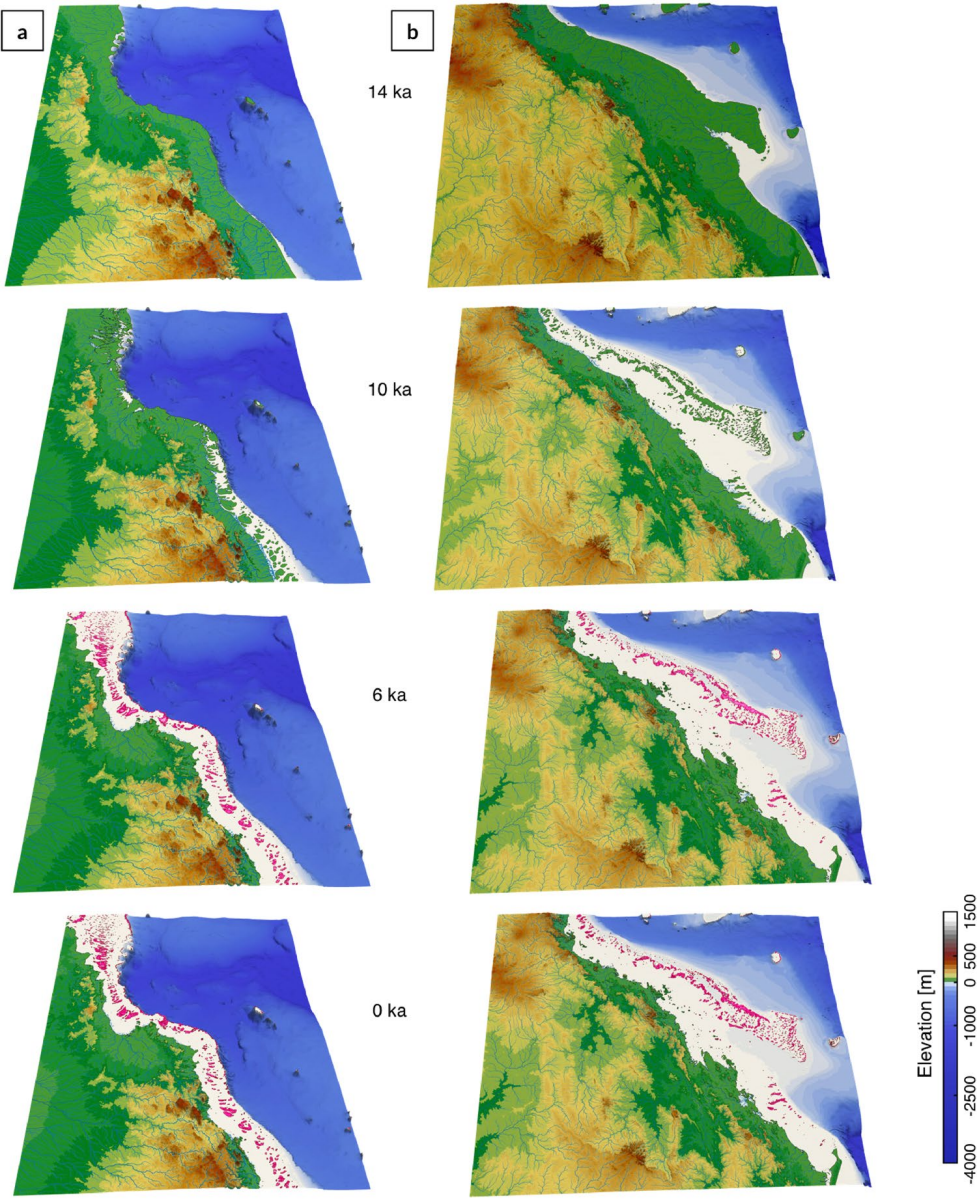
Model outputs for initial time step (14 ka), 10 ka, 6 ka and final step. Left and right panels show the evolution for the northern (**a**) and southern (**b**) parts of the GBR through the Holocene period (respectively i & ii regions in Fig. 1a). Pink color displays presence of coral reef at given time intervals. Model outputs are visualised using Paraview (V 5.2.0).

**Figure 4:**
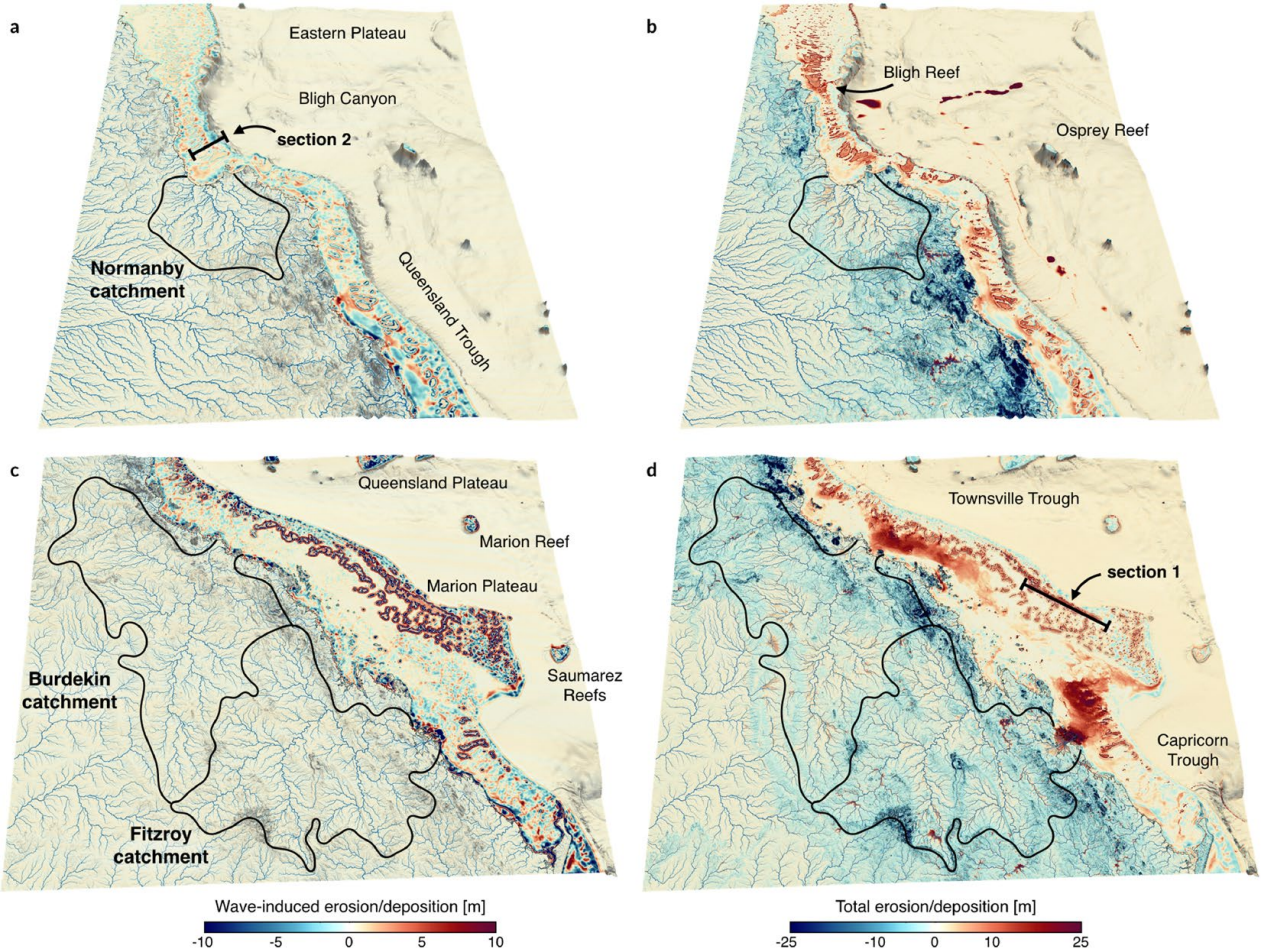
Maps of cumulative erosion/deposition in metres at final time step for the northern (a and b panels) and southern (**c** and **d**) GBR regions visualised using Paraview (V 5.2.0). Left panels (a and c) display the cumulative effects of wave-induced erosion and deposition over the simulated period (14 ka). Right panels (b and d) show the erosion, deposition and reef evolution for the 14 ka induced by the combination of fluvial and waves processes as well as reef growth. Sections 1 and 2 are the locations of the stratigraphic cross-sections displayed in Fig. 5.

**Figure 5:**
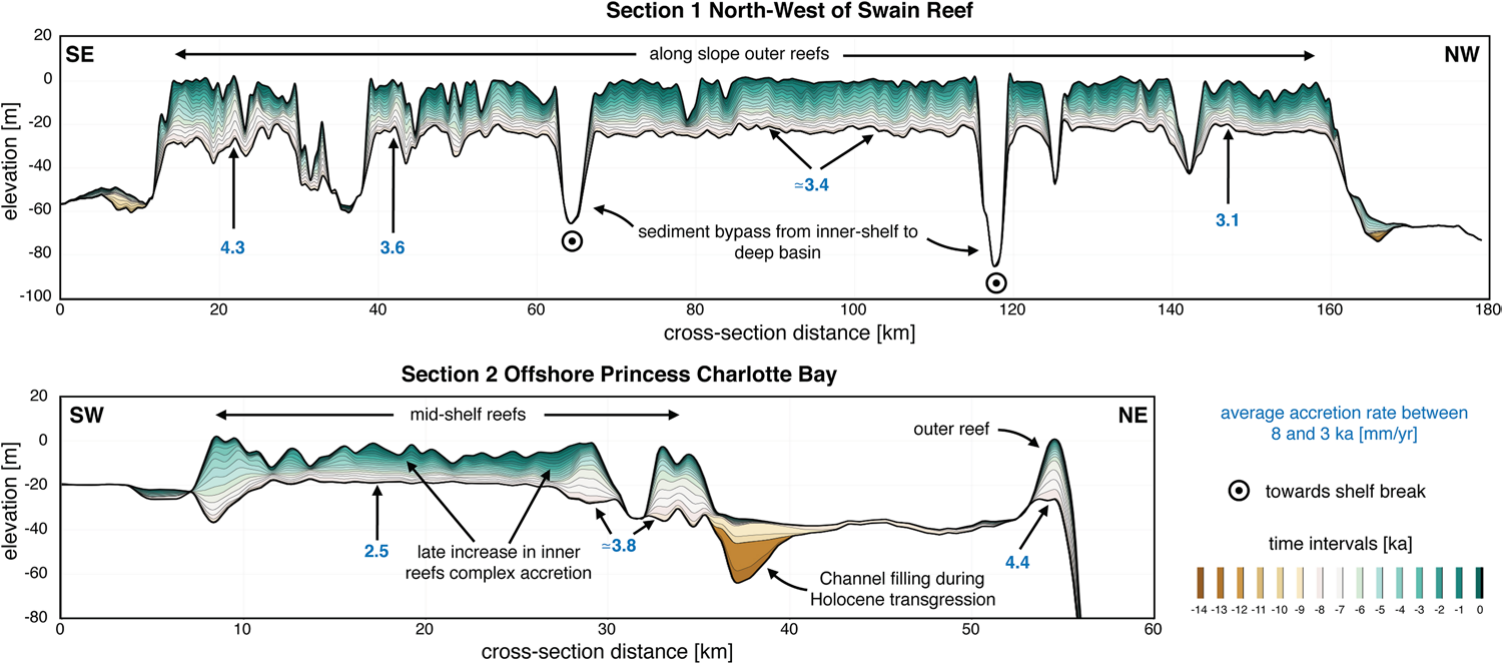
Cross sections through the model predicted stratigraphy showing time layers of mixed siliciclastic-carbonate accretion NW of Swain Reef and offshore of Princess Charlotte Bay (regional locations of these sections are presented in Fig. 4).

During the mid deglacial (14 ka), the exposed shelf acts as a bypass area where large river systems transport sediment directly to the shelf break (Fig. 3, 14 ka). Sediment transfer on the upper slope preferentially occurs through the numerous shelf incised submarine canyons and sediments accumulate at the base of the slope and along the basin troughs (especially on the northern GBR slopes (Fig. 4)). The rapid sea level transgression (prior to 8 ka) coincides with filling of previously incised channels and existing depressions (section 2 in Fig. 5). In northern GBR region, sediment transfer within the canyons persists and siliciclastic accumulation remains high in the deeper parts of the basin with a prominent submarine fan forming east of Bligh Reef and significant accumulation in the Bligh Canyon (Fig. 4b). In southern region (Fig. 4d), deposition is confined within the shelf with only hemipelagic deposition on the offshore basins. Differences in margin physiography (i.e. shelf width, slope gradient and canyon type) between the northern and southern portions of the GBR seem to be the main control affecting sediment dispersal within this period, which is in agreement with existing literature^58^.

From 8 to 2.5 ka, coral reef growth across the entire shelf coinciding with decrease in rate of sea level rise (Fig. 3, 6 ka). This period corresponds to conditions of higher rainfall and offshore wave energy; therefore, our model shows both an increase in terrigenous siliciclastic sediment delivered through riverine transport to the coast and a strong reworking of marine deposits by longshore drift on the inner shelf (Fig. 4a,c). A fine balance between climate, sea level and margin physiography enables coral reefs to thrive during this time interval. First, most of the delivered sediments remain in the coastal domain except in regions adjacent to the Fitzroy and Burdekin catchments (Fig. 4d). Second, the rate of sea level rise is slow enough to allow, in combination with higher wave energy, active coral reef growth. As shown in Fig. 5, from 8 to 3 ka, most reefs are able to catch-up with sea level rise, growing at rates faster than 4 mm/yr in some regions. The model also realistically simulates a time lag of approximately 1.8 ka between the initial flooding of the antecedent Pleistocene substrate (after 9 ka) and reef *turn-on*, occurring between 8 and 7 ka. We attribute this lag to the increase in sedimentation rates during the early stages of transgression. The simulated lag matches well with a documented lag of 0.7 to 2 ka after antecedent substrate flooding based on detailed radiometric dating of southern GBR drill cores^54^. Variations in reef accretion rates (Fig. 5) are largely controlled by paleo-surface elevation relative to sea level position and substrate composition. Wave energy is also playing an important role as shown in section 2 (offshore Princess Charlotte Bay) where the accretion rate decreases from the most energetic wave environments (4.4 mm/yr on the outer reef) to the more quiescent regions (2.5 mm/yr for the mid-shelf reefs).

During the late Holocene, sea level stabilisation and rainfall decrease, causing reduction in fluvial erosion and marine sediment accumulation in our model. Overall reef accretion rates drop significantly over the last 3 ka as they are limited by the lack of accommodation space. Despite a general reduction in vertical accretion, we note the increase of reef accretion rates in the mid-shelf reefs complex as shown in Fig. 5 (section 2). This corresponds to a phase of significant lateral reef accretion observed in a meta-analysis of all available GBR reef flat cores^59^ during this time interval that also represents the later stage of reef maturity in the Hopley’s classical genetic reef model^40^.

## Discussion

To assess the effectiveness of our approach we: (1) discuss the different components of our source-to-sink sediment transport model and; (2) compare the simulated sediment accumulation patterns across the GBR margin with conventional sedimentation models and key field observations.

Traditionally, most studies of sedimentation on continental margins have either been conducted in the context of a pure carbonate or a siliciclastic sedimentary system. Yet, these two systems respond very differently to sea level fluctuations. A classical idea suggests that maximum sediment accumulation on siliciclastic margins is associated with lowstands, when rivers incise the shelf and transport sediments to the continental slope and basin. Inversely, for carbonate margins, highest sediment accumulation is related to transgressions and highstands, when reefs are submerged and productive. In the context of tropical/subtropical mixed siliciclastic-carbonate systems, like the GBR, sediment accumulation has been described by the so-called reciprocal model^60^. In this model, the dominance of either terrigenous or biogenic accumulation is simply derived from the two end-member systems described above (either pure carbonate or pure siliciclastic systems) but recent observations have challenged this view^58,61^. From 14 to 12 ka BP (Fig. 3), sea level rose from about 80 to 60 m below present sea level. Our results show that prior to 14 ka, much of the continental shelf across the GBR was exposed, river mouths were close to the shelf-edge and rivers discharged siliciclastic material directly to the continental slope and deep basins. However, due to margin physiography, dominant waves direction and upstream catchments geometry, our model results exhibit some clear distinctions between the northern (Fig. 3a) and southern regions (Fig. 3b).

**Figure 6:**
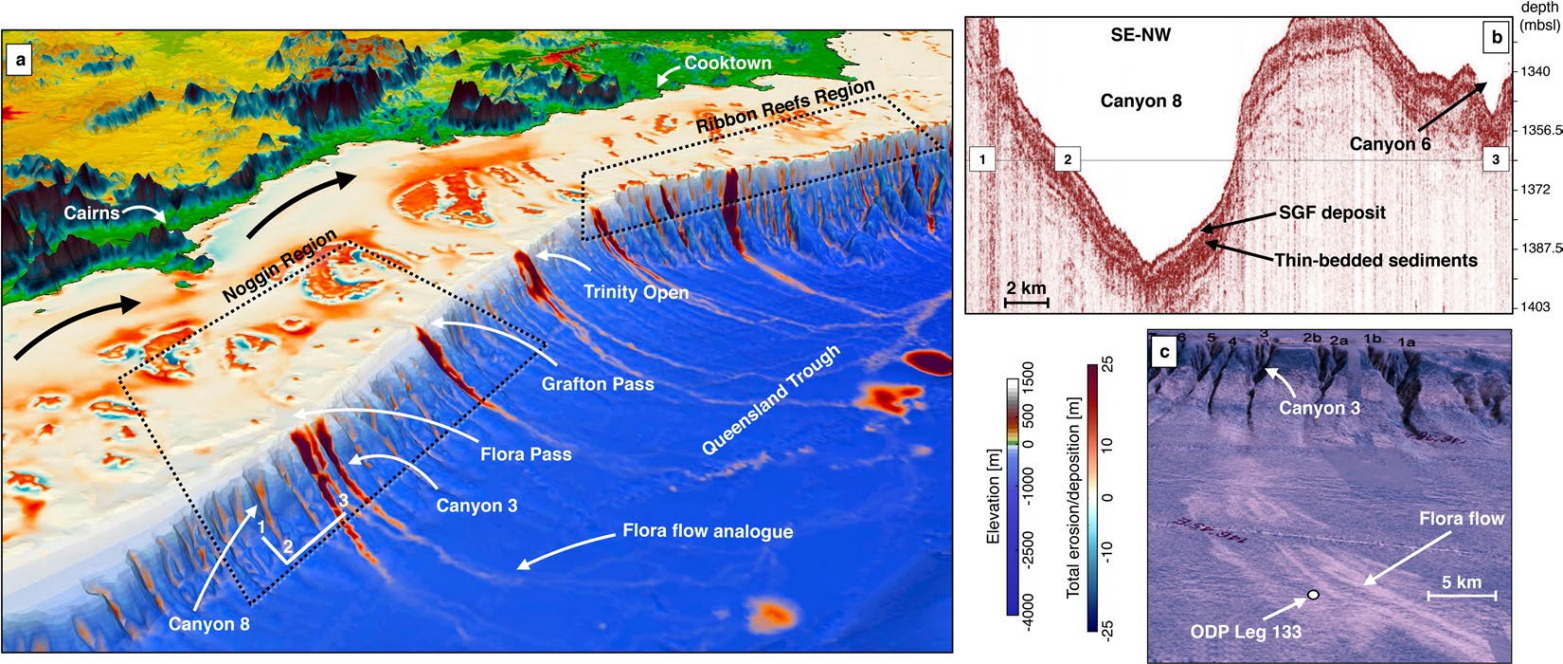
(**a**) 3D view of the GBR between Cairns and Cooktown highlighting sediment transport from mountain ranges to the coast, and from the coast to the Queensland Trough (generated using Paraview (V 5.2.0)). On the inner shelf dominant wave direction (SE) rework sediments to the North (black arrows). On the mid shelf, coral reef develops. Sediment transfer across the slope happens through V-shaped canyons. White lines (1–2–3) mark the location of seismic lines in b. (**b**) Topas seismic section illustrating sediment gravity flow (SGF) and thin-bedded deposits for Canyon 8. (**c**) 3D view of the Noggin region (canyons and slope) with draped GLORIA side-scan sonar backscatter imagery. High-reflectivity (white toned) areas correspond to SGFs and landslide deposits (**b** and **c** are adapted from Puga-Bernabéu *et al*.^58^).

In the north, filling of river channels by terrigenous sediments happens quickly during the early stage of the transgression (from 14 to 11 ka as shown in Fig. 5 section 2). Additionally, accumulation along the Bligh Canyon and the Queensland Trough remains high up to the late stage of the transgression (after 5 ka BP, Fig. 4b). This margin (Fig. 6a) is characterised by two climatic zones, the wet and the dry tropics, resulting from variability of seasonality and rainfall intensity^61^. Current sediment supplied to the shelf (~0.17 Mt/yr in the wet tropics and ~2.2 Mt/yr in the dry tropics) is controlled by several rivers draining from mountains and tablelands adjacent to the coast^25^. Our model shows that wave induced northward longshore transport redistributes: (1) terrigenous sediments in shallow water (<20 m) and (2) carbonate sediments around Holocene reefs mostly after the catch-up phase that ends around 3 ka. Sediment exchange between the shelf and the basin happens primarily through a series of shelf incised submarine canyons whose morphological characteristics have influenced the Holocene sedimentation dynamics^58^. Comparisons between the Noggin (NG) and the Ribbon Reefs (RR) regions show differences in sediment gravity flows deposition with significantly higher rates in the deeper basins facing the RR region. This result is consistent with previous studies in the area^13,62^, recording generally thicker and more frequent turbidites deposits in the RR canyons at this time. The modelled deposition thicknesses (ranging between 10 and 20 m–Fig. 6a) in the canyons along the NG region match with interpreted thicknesses obtained from Topas seismic section (Fig. 6b). We found that abundant sediment gravity flows have been deposited along the Queensland Trough during the Late Holocene, consistent with available subsurface data from the ODP Leg 133 (Site 823)^63^ (Fig. 6c). In our model, the depositional pattern in the trough agrees with sonar backscatter imagery (Fig. 6c) showing the Flora flow following the general slope direction which gently deepens towards the north. Previous studies of Site 823 cores confirm that the Flora flow has been a long-term site of significant coarse grained sedimentation, with over 2000 turbidites, debris-flow and slump deposited since the Miocene^63^. Through the transgression, the amount of material transported to the slope continuously declines and most of the terrigenous sediments accumulate on the middle and outer shelf. After 5 ka BP, during the late transgression phase and the sea level highstand, neritic carbonate production (Fig. 5 section 2) becomes the dominant source of sediment in the outer shelf, while terrigenous sediment is retained on the inner shelf close to the catchments outlets.

Conversely, in the south, simulated sediment accumulation along the continental slope and basin is marginal (Fig. 4d). From 14 to 12 ka BP, the Burdekin and Fitzroy rivers meander across the exposed continental shelf and disperse into a series of channels and large depressions as they approach the exposed reef platforms (Fig. 3b at 14 ka). Consequently, most of the siliciclastic sediments transported by these rivers accumulate on the continental shelf during lowstand, ponded behind the outer barrier fringing reefs (Fig. 3b at 10 ka). This result aligns well with the transgressive shedding model proposed for the Burdekin region based on a series of marine cores drilled along the margin in this area^12,61,64^. It highlights the complex relationship between paleo-surface morphology, barrier reef continuity, margin slopes and canyons geometry. Subsequently, during the transgression, the continental shelf is flooded by the rising sea, the dominant southeasterly offshore wave conditions favour sediment remobilisation by waves in this region (Fig. 4c). However, in our model, this remobilisation phase is not strong enough to transport significant amounts of sediment onto the continental slope. This result suggests that additional transport mechanisms such as tidal currents and/or cyclones might need to be incorporated to explain dominated turbidite deposition and hemipelagic sedimentation found on the south central GBR continental slope during the late transgression stage^65^.

In addition to the limitation mentioned above, simulated sediment erosion, transport and deposition as well as reef evolution could be further refined by incorporating additional processes such as karstification, cementation, mass wasting (i.e. slides and slumps), Halimeda bioherm development, tidal currents and wind induced wave generation but also extra factors influencing reef production and destruction based on changing conditions of temperature, salinity and water quality (in terms of nutrient influx) within the upper part of the water column. Nevertheless, in its current state, our framework already provides useful insights and quantitative metrics that could be used to better constrain the effects of deglacial to Holocene climatic variability on sediment dynamics in the GBR region^39,54,58,64^. The model demonstrates that sediment accumulation is a regional geological phenomenon and has played a significant role in controlling the distribution of coral reefs during the last sea level transgression. Over thousands of years, reduction in accommodation space, due to sea level stabilisation, has generated an increase in shelf sediment accumulation especially in the vicinity of large river systems (*e.g*. Burdekin and Fitzroy catchments, Fig. 4d). Future increase in sediment supply might result in the physical burial of inner-shelf reefs and, combined with resuspension and mobilisation of sediments by longshore drift, could also pose a significant threat to mid- and outer-shelf reefs. These predictions however will need to be balanced with projected rates of sea level rise^1^ that could increase accommodation space drastically, possibly causing: (1) restricted marine sediment accumulation to coastal domains and limited aggradation on the continental shelf; and (2) enhanced vertical reef accretion rates. Our model has the potential to quantitatively test these hypotheses in a consistent and efficient way and could be used to estimate the implications of long-term future climate predictions on the evolution of other mixed siliclastic-carbonate systems.

In this paper, we present a unified framework to simulate sediment transport dynamics from source to sink. The approach falls into the so-called reduced-complexity family of models in that it relies on simple, though not simplistic physics to drive fluid motion and associated sediment transport by rivers and waves. Modelled changes in accommodation space, wave energy and sedimentation rates depend on forcing conditions (*e.g*. rainfall, tectonics, sea level) which could favour the development of coral reefs simulated based on fuzzy logic rules. By efficiently linking different components of the Earth system over geological times, our model represents a major improvement to existing forward models as it allows high resolution simulation at regional to continental scales, thereby enabling precise comparisons between field observations and models. It also constitutes a complementary approach to resource intensive climate models often limited to decadal to centennial evolution which might be too short to investigate the resilience of some of the studied processes especially in the case of mixed siliciclastic-carbonate systems where many reef colonies lifetime can span over more than 500 years. This study indicates that changes in runoff, sea level, sediment yield and wave energy have profoundly affected the past evolution of the GBR not only in regards to reefs evolution but also sediment fate from source-to-sink. By validating model results against geological observations, the approach could help to predict and quantify the impacts that will likely occur under changing climate, and needs to be considered in future ocean resources management and land use management. The numerical framework proposed in this study is widely applicable to other sedimentary regions (silicilastic or carbonate or mixed siliclastic-carbonate systems), especially those that are data-scarce.

The results and data presented and discussed here were generated using Badlands model. Supporting information has been made available through Github at GBR-input (https://github.com/badlands-model/pyBadlands-Published/tree/master/NatSciRep-GBR) and contain all the input and forcing conditions files required to reproduce the experiments published in this article. All the model outputs were visualised using the Paraview software (V 5.2.0) from Kitware, Sandia National Labs and CSimSoft.
